# The effects of hydroxychloroquine on pregnancy outcomes in infertile women: a systematic review and meta-analysis

**DOI:** 10.25122/jml-2022-0095

**Published:** 2023-02

**Authors:** Maryam Mirzaei, Sara Amirajam, Elham Sadeghi Moghimi, Soudabeh Behzadi, Abbas Rohani, Nasibeh Zerangian, Neda Khalili Samani, Simin Soudagar, Masumeh Ghazanfarpour

**Affiliations:** 1 Department of Obstetrics and Gynecology, Faculty of Medicine, Jiroft University of Medical Sciences, Jiroft, Iran; 2 Avicenna Infertility Clinic, Avicenna Research Institute, Academic Center for Education, Culture and Research (ACECR), Tehran, Iran; 3 Department of Community Health Nursing, Vali Asr Nursery, Shiraz, Iran; 4 Student Research Committee, School of Nursing and Midwifery, Shiraz University of Medical Sciences, Shiraz, Iran; 5 Department of Educational Psychology, Islamic Azad University Mobarakeh Branch, Esfahan, Iran; 6 School of Public Health, Iran University of Medical Sciences, Tehran, Iran; 7 School of Nursing and Midwifery, Isfahan University of Medical Sciences, Isfahan, Iran; 8 Social Security Organization, Isfahan University of Medical Sciences, Isfahan, Iran; 9 Department of Nursing, Faculty of Nursing and Midwifery, Hormozgan University of Medical Sciences, Bandar Abbas, Iran; 10 Student Research Committee, Kerman University of Medical Sciences, Kerman, Iran

**Keywords:** Hydroxychloroquine, pregnancy, infertile, systematic review

## Abstract

A promising strategy for controlling repeated implantation failure (RIF) may be the use of hydroxychloroquine (HCQ). To the best of our knowledge, no systematic review has been conducted on the effects of hydroxychloroquine on pregnancy outcomes. A systematic research of the following electronic databases was conducted: Cochrane, EMBASE-Ovid, PubMed, Web of Science, and Scopus from inception to December 2021, using the following keywords [hydroxychloroquine] AND [infertility]. Fertilization and rate of live birth were significantly higher in the HCQ+ prednisone (PDN) group than in the PDN alone group. However, the abortion rate was not different between the two groups. The meta-analysis of two studies revealed no statistical significance between the PDN group and HCQ+PDN group regarding clinical pregnancy rate (OR=.14 [95%CI: 0.4–4.370]; heterogeneity; P=0.13; I2=54%; random effect model) and implantation rate (OR=1.99 [95%CI: 0.94–4.2]; heterogeneity; P=0.37; I2=0%; fixed-effect model). While HCQ may help improve fertilization and live birth rates, adding it to prednisone did not improve overall pregnancy outcomes. This systematic review should be used with caution due to the small size, study design, and difference in the studies' population

## INTRODUCTION

Infertility is regarded as one of the global reproductive health problems, with a prevalence of 8–15% [1]. Assisted reproductive techniques (ARTs) are a range of medical interventions that offer a solution for couples facing infertility challenges [1]. Despite significant advances in ART to date, evidence suggests that the current implantation rate remains at 30%. Additionally, there are ongoing challenges in addressing female fertility concerns related to implantation failure [2].

Repeated implantation failure (RIF) is a failed clinical pregnancy following the transfer of a minimum of four embryos of good quality in at least three frozen or fresh cycles to a woman under 40 years of age [3]. Recent studies have shown that RIF is associated with impaired innate or acquired immune responses, so the immunological factors can cause implantation failure by decreasing endometrial receptivity [4]. During pregnancy, the immune system holds a critical function that can both aid in the normal progression of fetal development and cause complications. Successful pregnancy occurs by balancing the cytokines Th1, Th2, and Th17 and the proper functioning of regulatory T cells, Tregs [5].

Few treatment suggestions exist for dealing with RIF challenge, like sequential embryo transfer of cleavage stage embryos and blastocysts, intratubal transfer of zygotes and embryos, blastocyst transfer preimplantation genetic screening for aneuploidy screening, and immunological tests [6]. Therefore, a promising strategy for controlling RIF could be to regulate or suppress the immune system by applying immunosuppressants or immunomodulators [7, 8].

Immunoregulatory therapy has the potential to be a promising method for managing immune system disorders. Cyclosporine (CsA), hydroxychloroquine (HCQ), or prednisone (PDN) could reportedly impede the expression of Th-1 cytokines, elevate the count of Tregs and trigger maternofetal tolerance [9].

The antimalarial hydroxychloroquine (HCQ) is used for reducing immunologic base infertilities [10], and it is widely administered in people suffering from various autoimmune disorders, especially systemic lupus erythematosus (SLE). Recent advances in the therapeutic landscape of HCQ have been made possible by a deeper understanding of its mechanisms of action [11].

Various immunomodulatory and anti-inflammatory properties of hydroxychloroquine have been reported, including increased Treg proliferation, reduction in complement-mediated antigen-antibody reactions, suppression of pro-inflammatory cytokines (like TNF-α, IL-6, IL-17, IFN-γ, and IFN-α), stabilization of lysosomal membranes, and inhibition of phospholipase [4]. In both *in vitro* and SLE individuals, hydroxychloroquine could suppress the IL-17 expression and the Th17 cell differentiation [12]. The administration of immunomodulators before embryo transfer can improve the outcome of the IVF process. Some researchers have studied the combination of PDN+HCQ immunomodulatory drugs [9, 13]. The findings are contradictory, probably because such studies did not examine selected patients with an immunodeficiency that may have increased the Th1/Th2 ratio [9]. To the best of our knowledge, no systematic review has been conducted to evaluate the effects of hydroxychloroquine on pregnancy outcomes.

## MATERIAL AND METHODS

### Data collection tools

**Figure 1:**
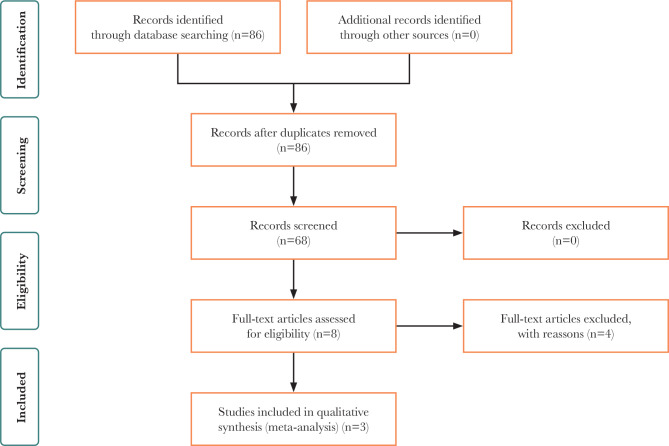
PRISMA flowchart.

The search strategy for collecting required data was performed on electronic databases, including Cochrane, PubMed, Web of Science, Scopus, and EMBASE-Ovid from inception to December 1, 2021, using the main keywords [hydroxychloroquine] AND [infertility] (Figure 1). The study population was pregnant women, and the inclusion criteria consisted of all clinical trials evaluating the efficacy of hydroxychloroquine in women who experienced RIF.

### The process of selecting related articles

Two separate reviewers selected the relevant articles during two phases. The first step was the screening process, where they reviewed the title of articles for eligibility. In cases of ambiguity during the article selection process, the reviewers assessed the compliance of the title and abstract with the established inclusion and exclusion criteria to ensure accuracy. Suspicious cases that needed to be read in full text to make a decision entered the second phase. The second step was to review the full text of screened articles. Articles that ultimately met the eligibility criteria were enrolled in the systematic review. The search was completed with a thorough analysis of all enrolled articles, review articles, and article references on the subject under study.

### Data extraction

Two separate researchers reviewed all searched articles and extracted and recorded the required data in a pre-designed table (Table 1) containing the name of authors, type of intervention, duration of intervention, inclusion criteria, specification of control, years of publication, sample size, results, main outcomes, and complications.

**Table 1:** Demographic and clinical characteristics of the studies included in the systematic review.

Author/year/country	Type of study	Age	Outcome	Number of subjects	Intervention (dose and duration of treatment)	Comparison (dose and duration of treatment)	Inclusion criteria	Results	Quality of study
Meng *et al*., 2020 China,	Retrospective cohort study	34	-	PDN+HCQ+CsA/PDN+HCQ/PDN=21/9/11 in treated group/control group n=30	PDN+HCQ+CsA/PDN+HCQ/PDN	Treated Nontreated	IVF-ET with increasing Th1/Th2 ratio	Rate of live birth (P=0.026). Rate of biochemical pregnancy (P=0.18), Implantation rate (P=0.15), clinical pregnancy (P=0.0743) higher than control group but no statistical significance.	7
Lian *et al*., 2020, China	Retrospectively	-	Implantation rate, fertilization rate, clinical pregnancy rate, and abortion rate	Prednisone group n=65/prednisone+ HCQ group n=91	Prednisone (7.5 mg/)+HCQ (0.2), twice a day	Prednisone (7.5 mg) twice a day	Subjects that underwent an IVF-ET with positive ANA+/anti-dsDNA	A significant improvement in fertilization rate(p=0.017), implantation rate(p=0.032), clinical pregnancy rate (p=0.028) in the prednisone +HCQ group than alone prednisone group	7
Sadeghpour *et al*., 2020, Iran	Clinical Trials (before/after)	33.6±4.2	Biochemical pregnancy	60	400 mg HCQ	Daily for 16 consecutive days.	-	Comparison of before with after treatment with HCQ did not significantly change the biochemical	

PDN – prednisone; HCQ – hydroxychloroquine; CsA – cyclosporine.

### Risk of bias in included articles

The methodological quality of cohort studies was reported according to the modified Newcastle-Ottawa scale, which consists of three factors: assessment of outcome, patient selection, and A score ranging from 0–9.

### Statistical analysis

The comprehensive meta-analysis (CMA) software version 2 was used for data analysis. I2 and Q Cochran test as the heterogeneity index was also reported. A random-effect model was used instead of the fixed-effect model to compare the two groups.

According to Higgins *et al*. [14], the values of <25%, 25–75%, and >75% indicate low, moderate, and high levels of heterogeneity, respectively. The effect of hydroxychloroquine on pregnancy outcome was investigated using a forest plot. The square size indicates the number of samples in each study, and the lines drawn on either side show a 95% confidence interval (95% CI) for the impact of each study.

## RESULTS

### Abortion rate

Patients in the prednisone+HCQ group reported a lower abortion rate (7%) than the alone prednisone group (12.9%). However, there was no significant difference between groups of patients with ANA and ds-DNA positive [15].

### Rate of live birth

Meng *et al*. examined retrospectively the subjects who had failed IVF-ET and increased the Th1/Th2 ratio from 1/2019 to 3/2020. The highest rate of live birth was observed in the hydroxychloroquine (HCQ) + cyclosporine (CsA) + prednisone (PDN) group (52.4%), followed by PDN+HCQ group (33.3%), PDN group (27.3%) and untreated group (16.7%). A significant difference among groups PDN+HCQ+CsA/PDN+HCQ/PDN=21/9/11 was reported (P=0.03) [9].

### Fertilization rate

In a retrospective study, Lian *et al*. in south China examined participants (n=156) under treatment for IVF-ET, who were positive for ds-DNA and ANA with no symptoms, between January 2010 and December 2016. Patients with ANA and ds-DNA positive in the prednisone + HCQ group showed a higher fertilization rate (75.8%) than the alone prednisone group (60.0%), with a significant difference between groups (p=0.017) [15].

### Rate of biochemical pregnancy

The highest rate of biochemical pregnancy in the Meng *et al*. study was observed in the PDN+ HCQ+CsA group (66.7%), followed by alone prednisone (54.5%), untreated group (40%), and PDN+HCQ group (33.3%) but there was no statistical significance among PDN+HCQ+CsA/PDN+HCQ/PDN=21/9/11 groups (P=0.18) in patients with increased peripheral blood Th1/Th2 ratios [9].

In the study of Sadeghpour *et al*., the RIF group was treated with oral HCQ (n=60) on the fourth day of menstruation until the twentieth day of menstruation and two days prior to embryo transfer until the day of the pregnancy test, in total, 16 days in another cycle. The rate of biochemical pregnancies using HCQ was 23.3%, while 76.7% of the patients experienced no pregnancy. Before and after treatment with HCQ did not significantly change the biochemical pregnancy rate in RIF patients [16].

### Implantation rate

The highest implantation rate was in the PDN+HCQ+ CsA group (45.9%), followed by the prednisone+HCQ group (31.3%), prednisone group (33.3%), and untreated group (23.9%). However, no statistical significance was reported among PDN+ HCQ+CsA/PDN+HCQ/PDN=21/9/11 groups (P=0.15) in patients with increased peripheral blood Th1/Th2 ratios [9].

In Lian *et al*. study, patients in the PDN+HCQ group (29.7%) showed a significantly higher implantation rate than the PDN group (15.4%), with a significant difference between groups (p=0.03) in ANA and ds-DNA positive patients [15].

**Figure 2:**
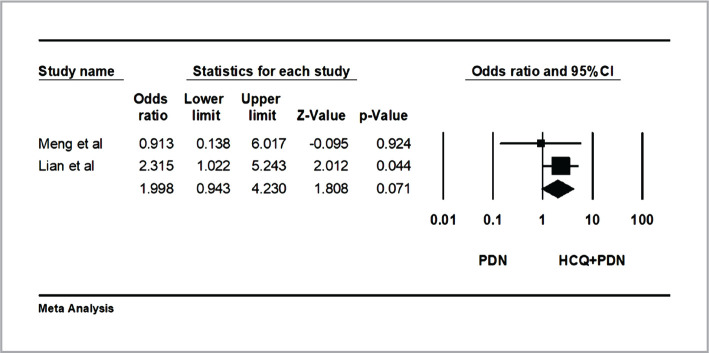
Effects of hydroxychloroquine on implantation rate. The horizontal lines denote the 95% CI; ♦ – combined overall effect of treatment.

Combining the results of the two studies [9, 14] in a meta-analysis revealed no statistical significance between the PDN group and HCQ+PDN group regarding implantation rate (OR=.99 95% CI: 0.94–4.2; heterogeneity; P=0.37; I2=0%; fixed effect model) (Figure 2).

### Clinical pregnancy

**Figure 3:**
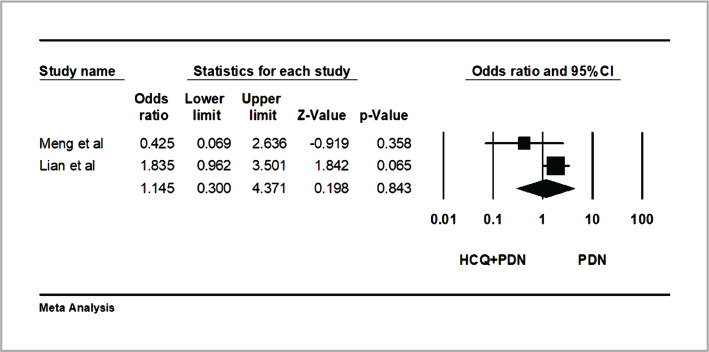
Effects of hydroxychloroquine on clinical pregnancy rate. The horizontal lines denote the 95% CI; ♦ – combined overall effect of treatment.

In the first study, the highest rate of clinical pregnancy rate was in the PDN+HCQ+CsA group (57.1%), followed by the PDN group (54.5%), HCQ+PDN group (33.3%) and the untreated group (30%). However, there was no significant difference between the PDN+HCQ+CsA/PDN+HCQ/PDN=21/9/11 groups (P=0.07) in the subjects with elevated Th1/Th2 ratio in peripheral blood [9]. In the second study, patients in the PDN+ HCQ group (62.6%) reported a significantly higher clinical pregnancy rate than in the PDN group (47.7%), with a significant difference between groups (p=0.02) in ANA and ds-DNA positive patients [15]. Combining the results of the two studies [9, 14] in a meta-analysis revealed no statistical significance between the PDN group and HCQ+PDN group regarding clinical pregnancy rate (OR=1.14 (95% CI: 0.4–4.370); heterogeneity; P=0.13; I2=54%; random effect model (Figure 3).

## DISCUSSION

As far as we know, there is no systematic review of the effects of hydroxychloroquine (HCQ) on pregnancy outcomes. The prednisone + HCQ group showed a significantly increased fertility rate compared to the prednisone group [15]. Meng *et al*. did not detect any difference in clinical pregnancy, biochemical implantation markers, and live birth rate between the treated groups (PDN+HCQ+CsA), (PDN+HCQ), (PDN), and untreated groups [9].

Their findings contrast with other studies [16] for several reasons outlined below [9]. First, women with severe infertility were positioned on their list since the mean ratio of Th1/Th2 (TNFα/IL-4) was greater than the ratio of Th1/Th2 in infertile women and women with IVF failure. Second, the design was a retrospective cohort study [9] which differed from the study populations in other studies.

A higher rate of live births (P=.05) was reported in the group treated with hydroxychloroquine (67%) compared with the group without HCQ (57%) in women with antiphospholipid antibodies [13]. HCQ administration can have some benefits on RIF, such as few side effects and cost-effectiveness. Concerning the potential side effects of children exposed in utero, a systematic review showed a low risk of vision problems [17]. A recent meta-analysis indicated no increased risk in rates of major congenital, craniofacial, cardiovascular, genitourinary, or nervous system malformations [18]. Furthermore, HCQ administration has not been shown to increase the risk of premature birth defects or fetal death or to reduce live births in women suffering from autoimmune disorders [19].

In a study by Ghasemnejad-Berenji *et al*., the effects of hydroxychloroquine (HCQ) on the balance of Th1/Th2 cells were examined in 17 women with a history of recurrent implantation failure and elevated TNFα/IL-10 ratio (TNFα/IL10≥30.6). The results showed that HCQ treatment led to a downregulation of T-bet, a Th1 transcription factor, and an upregulation of GATA-3, a Th2 transcription factor. The study also revealed a significant increase in IL-4 and IL-10 (p<0.05) and a significant decrease in IFN-γ and TNFα (p<0.05), indicating that HCQ may have a beneficial effect on shifting the Th1/Th2 balance towards a more favorable Th2-dominant state in women with RIF [20]. According to Sadeghpour *et al*., HCQ could down-regulate RORγt (the Th17 transcription factor), the expression and function of Th17-related cytokines, and up-regulate FOXP-3 (the T-reg transcription factor), and the expression and function of Treg related cytokines (P<0.001) [10]. Successful pregnancy outcomes may be linked to several factors, including the balance of Th1 and Th2 cells in the immune system. High levels of Th1 cell proliferation may lead to embryo rejection, while high levels of Th2 cell proliferation can result in the production of pregnancy cytokines such as TNF-α, which can inhibit trophoblastic growth and increase inflammation and thrombosis in the blood vessels of the uterus. On the other hand, cytokines produced by Th2 cells, such as IL-10, IL-6, and IL-4, can suppress the tissue factor secreted by Th1 cells through monocytes, potentially promoting successful implantation and pregnancy [21]. Additionally, the expression of both IL-17 and RORγt decreased following HCQ therapy in endometrial tissue samples obtained from patients with RIF diagnosed with cell-mediated autoimmune disorders. Furthermore, the expression of IL-10, TGF-β, and Foxp3 increased following HCQ therapy.

Moreover, the HCQ therapy in RIF pregnant women suffering from cell-mediated autoimmune disorders impacts the endometrial tissue and peripheral blood Th17/Treg ratio, reduces Th17 activity, and increases Treg expression. Successful pregnancy outcomes can be achieved by the HCQ immunomodulatory behavior towards Th17 imbalance and Th17 and Treg imbalance. In third place, some immunological causes of RIF can be affected by the medicinal features of HCQ, such as immunomodulatory, anti-thrombotic, and vascular protective activities [16].

The generalizability of the findings from this systematic review to the broader population of women with RIF should be approached with caution due to limitations in the study design. Specifically, in the study by Meng *et al*., the mean IVF-ET cycle was 2.1, which may not be sufficient for a definitive diagnosis of RIF [9]. Additionally, the sample size in the study was small, further reducing the robustness of the findings.

## CONCLUSION

The administration of hydroxychloroquine (HCQ) may have a potential impact on enhancing the outcomes of embryo transfer, such as live birth rates and fertilization rates. Nevertheless, it is important to approach these findings with a degree of caution due to the limitations in sample size, study design, and differences in the population of the studies.
